# 《胸部恶性肿瘤围术期静脉血栓栓塞症预防中国专家共识（2018版）》解读之流行病学篇

**DOI:** 10.3779/j.issn.1009-3419.2019.12.01

**Published:** 2019-12-20

**Authors:** 益鑫 孙, 辉 李

**Affiliations:** 100020 北京，首都医科大学附属北京朝阳医院胸外科 Department of Thoracic Surgery, Beijing Chaoyang Hospital, Capital Medical University, Beijing 100020, China

**Keywords:** 静脉血栓栓塞症, 流行病学, 胸外科手术, 围术期, Venous thromboembolism, Epidemiology, Thoracic surgery, Perioperative period

## Abstract

胸部恶性肿瘤患者围术期静脉血栓栓塞症（venous thromboembolism, VTE）是一种需要引起重视的胸外科围术期并发症，中国胸外科静脉血栓栓塞研究协作组针对胸部恶性肿瘤患者围术期VTE的预防，发布了国际首部《胸部恶性肿瘤围术期静脉血栓栓塞症预防中国专家共识》（2018版）。本文将对其中胸部恶性肿瘤围术期VTE的流行病学特征、国内外面临的挑战及预防现状进行解读，以助于更好地理解共识相关内容。

## Abstract (en)

Perioperative venous thromboembolism (VTE) of patients after thoracic cancer surgery has proved to be a serious or even fatal postoperative complication, which calls for great attention. China National Research Collaborative Group released the first edition of Chinese experts consensus on the perioperative VTE prophylaxis in 2018. This article is to interpret it on the aspect of epidemiological characteristics of perioperative venous thromboembolism and the current status of prophylaxis in China and other countries, in order to provide a better understanding of Chinese experts consensus for readers.

静脉血栓栓塞症（venous thromboembolism, VTE）是外科围术期影响预后的严重并发症之一。2018年，中国胸外科静脉血栓栓塞研究协作组发布了首部《胸部恶性肿瘤围术期静脉血栓栓塞症预防中国专家共识》（2018版）^[1]^（以下简称《共识》），填补了我国胸外科在恶性肿瘤围手术期VTE预防方面缺乏指南共识类文件的空白，通过规范胸外科恶性肿瘤患者围手术期VTE的预防治疗措施，从而达到降低VTE发病率的目的。《共识》指出，我国胸外科恶性肿瘤患者面临着产生围手术期VTE的严峻挑战，而大多数中国胸外科医生更是存在着对VTE认知度与关注度不足的问题，在VTE防治方面，情况不容乐观。本文将围绕胸部恶性肿瘤围手术期VTE的流行病学特征进行文献回顾，并简要介绍国内外在预防VTE中面临的挑战与防治现状，以增进胸外科医生对《共识》相关内容的理解，提高对预防胸部恶性肿瘤围手术期VTE的重视程度。

## 1 VTE流行病学特征

### 1.1 VTE总体患病率

一项涉及到1, 270万人口的回顾性研究^[2]^显示，VTE在全美范围内的患病率在2006年达到了422/10万。另一项持续25年针对当地人群进行长期观察的队列研究^[3]^发现，得益于检测技术的进步以及医师的重视程度，致死性肺栓塞（pulmonary embolism, PE）的患病率有所下降，而深静脉血栓形成（deep vein thrombosis, DVT）的患病率则有所上升。

### 1.2 胸部恶性肿瘤围术期VTE发生率

早在19世纪，便已有相关论著描述恶性肿瘤患者的血液中呈现一种高凝状态。文献^[4]^已证实，恶性肿瘤与VTE的发生密切相关。

VTE作为一种严重影响恶性肿瘤患者生活质量与预后生存的并发症，可使患者的住院时间延长3倍，并增加医疗支出总费用^[5]^。同时影响患者的短期与长期生存，是接受肿瘤切除术后患者在30 d内死亡的最主要因素^[6-8]^，也是罹患恶性肿瘤患者的第二大死亡原因^[8, 9]^。

《共识》指出，胸部恶性肿瘤患者中多存在凝血机制异常，具有慢性弥漫性血管内凝血，肿瘤本身亦可分泌促凝物质，而被浸润的血管内膜则损失了相应抗血栓的能力，这些均为恶性肿瘤患者VTE高发的病理生理基础。相关队列研究^[10]^表明，恶性肿瘤患者发生VTE的风险为正常人群的20倍左右。一项纳入了91, 933例新诊断肺癌患者的流行病学研究^[11]^发现，新诊断肺癌患者在1年内VTE累积发生率约3%，2年内VTE累积发生率约3.4%。并呈现出非小细胞肺癌患者高于小细胞肺癌患者、腺癌患者高于鳞癌患者^[10-13]^、伴远处转移患者高于局限早期患者^[10, 11, 14, 15]^以及全肺及肺叶切除患者高于肺段切除患者^[16]^的特点。有相关报道^[2, 11]^证实，人群中肺癌患者罹患VTE的患病率约61/10万，并呈上升趋势，日益成为政府与社会卫生经济支出的一项重要负担。

除外肿瘤因素与患者自身因素，手术^[17]^、放化疗^[18, 19]^、靶向药物及抗血管生成药物^[6]^等肿瘤治疗手段，由于引起相应的血液动力学改变及可能造成的血管内皮损伤，均增加了VTE的发病风险^[6]^。美国临床肿瘤学会（American Society of Clinical Oncology, ASCO）《肿瘤患者静脉血栓栓塞防治指南》第1版指出，肺癌患者接受外科手术，术后DVT风险较非肺癌手术患者增加2倍，术后致死性PE风险增加3倍^[20]^。White等^[21]^分析了接受76类手术的1, 653, 275例患者，发现恶性肿瘤患者术后发生VTE的风险为对照组的1.7倍。Agzarian^[22]^的研究发现，因肺部恶性肿瘤接受肺叶、肺段、楔形、气管及全肺切除等术后患者，VTE总发生率约12.1%。Agnelli等^[23]^收集了2, 373例接受各种不同部位肿瘤切除术后患者，发现胸部恶性肿瘤切除术后VTE发生率约5%左右。Molena等^[24]^回顾了74, 361例恶性肿瘤术后患者，其中肺癌患者术后VTE发生率约1.8%。一项针对44, 656例肿瘤切除术后患者的多中心研究^[7]^报道，肺癌患者术后VTE发生率约2.7%，PE及DVT发生率均为1.4%左右。Christensen等^[25]^在一项荟萃分析中系统回顾了按标准纳入的19篇相关文章，认为接受手术切除后的肺癌患者中，VTE发生率约2%（范围0.2%-19%），且主要发生于围术期。

国内方面，中国临床肿瘤学会（Chinese Society of Clinical Oncology, CSCO）2015版《肿瘤相关VTE预防与治疗中国专家指南》指出，若无有效的预防措施，因肿瘤而行外科手术患者中，DVT和下肢近端DVT的发生率分别高达40%-80%和10%-20%，PE发生率为4%-10%^[26]^。我国一项流行病学研究^[27]^观察了673例接受手术、化疗及放疗的新发肺癌住院患者，发现VTE发生率约13.2%，其中DVT单独发生率约6.2%，PE单独发生率约4.9%，DVT合并PE发生率约2.1%。来自上海肺科医院的数据^[6]^显示，1, 001例肺癌患者在术后1个月、3个月、6个月、12个月和30个月VTE发生率分别为2%、3%、4%、5%和5.3%。在一项近期发表的中国单中心前瞻性队列研究^[28]^中，未经治疗的胸外科术后患者，VTE总发生率约13.9%，肺癌患者术后VTE发生率高达16.4%。该课题组随后进行的另一项回顾性研究^[29]^显示：在339例肺癌切除术后患者中VTE发生率约11.5%，若经术前下肢血管超声发现下肢肌间静脉扩张，则也为肺癌术后合并VTE的独立危险因素。《共识》认为，肺癌术后VTE的发生率约为7.3%-13.9%。

目前关于食管癌患者发生VTE的流行病学研究较为有限，研究报道围术期VTE发生率约为5%-14%。一项研究9种恶性肿瘤患者术后深静脉血栓情况的多中心研究^[7]^共纳入了44, 656例患者，其中共2, 093例食管胃肿瘤患者，统计发现食管胃肿瘤患者术后VTE发生率约4.2%，在统计的9种肿瘤患者中发生率最高。另一项由美国外科医师学会在2015年进行的大型回顾性研究^[24]^发现，在3, 126例食管恶性肿瘤患者中，术后VTE发生率约为5.9%，在所统计的5种恶性肿瘤中同样最高。

《共识》指出肿瘤治疗史为围术期VTE危险因素之一。对于行新辅助化疗后接受手术的肺癌患者，围术期VTE发生率未有准确报道。但普遍观点认为，曾接受化疗的胸部恶性肿瘤患者中VTE发生率更高^[14, 19, 30-32]^，在Khorana建立的适用于评估化疗患者VTE的风险模型中，化疗为一项明确的独立危险因素^[30]^。美国国家综合癌症网络（National Comprehensive Cancer Network, NCCN）2017版《癌症相关静脉血栓栓塞指南》则提供了细胞毒性药物、内分泌治疗及抗血管生成药物等3类药物可增加VTE风险的证据^[33]^。

在首次接受化疗的恶性肿瘤住院患者中，肺癌患者VTE发生率约为7%，食管癌患者VTE发生率约为6.72%^[34]^，已有的大型回顾性研究^[18, 35]^显示，肺癌患者在化疗开始后的1年内，VTE累积发生率为12.6%-13.9%，系统化疗可增加约6倍-7倍VTE发生风险，原因可能同化疗药物作用于血管内皮细胞并造成破坏相关。因此，对接受新辅助化疗等肿瘤治疗后进行手术的胸部恶性肿瘤患者，应加以重视。

### 1.3 围术期VTE死亡率

胸部恶性肿瘤患者术后合并VTE后，死亡率可陡然上升。根据Merkow等^[7]^进行的大型多中心回顾性研究发现，恶性肿瘤患者术后一旦合并VTE，死亡率从1.3%上升至8.0%。Kalweit等^[36]^研究了125例在施行肺部切除手术后早期死亡的患者，其中32例患者死于急性心肺循环功能衰竭，对其中19例死亡患者的尸检证实了致死性PE的存在，共占术后早期死亡患者的15.2%（19/125）。Trinh等^[37]^回顾了8种不同种类实体肿瘤术后的患者共2, 508, 916例，发现肺癌术后发生VTE的患者的死亡风险为未发生VTE患者的8.7倍，死亡率约为19.8%；食管癌术后发生VTE的患者，死亡率约13.6%，而未发生VTE的食管癌术后患者，死亡率仅为6.9%。

### 1.4 围术期VTE出现的时间分布

关于围术期VTE发生时间，已有大量研究及指南意见。研究^[6-8]^证实，围术期相关VTE事件在术后30 d内高发，其中致死性与症状性PE常出现于患者术后首次下床活动时，表现为呼吸困难、晕厥及心跳骤停，并伴随呼吸、心率、血压的急剧变化^[38]^，无症状性VTE则常常出现较为隐匿。Daddi等^[39]^指出，大部分围术期VTE事件为无症状性或症状表现不明显。

Mason等^[40]^的研究证实，肺癌患者围术期VTE的发生在术后第7天到达高峰，随后出现下降，在术后第35-40天恢复术前水平，与患者出院时间分布曲线相重合，同时大部分VTE事件发生在患者出院之后。Merkow等^[7]^报道，发生在出院后的恶性肿瘤术后患者VTE事件约占总体的33.4%。Agneli等^[23]^的研究发现，近半数的肿瘤术后VTE发生于患者接受手术21 d后。White等^[21]^的研究表明，约56%的术后VTE事件发生于患者离院后，并与Alsubaie等^[41]^的结果一致，观察到了术后90 d时间跨度内的VTE事件。Bustos等^[42]^同样佐证了约54%的围术期VTE患者于出院后期间发病。现已有多项研究^[43-45]^表明，延长抗凝预防时间可降低出院后患者的VTE发生率：一项术后随访时间长达3个月的双盲随机对照试验，比较了恶性肿瘤术后抗凝28 d组与术后抗凝7 d组之间的差异，结果显示延长抗凝策略可将VTE的发生率从12.0%降至4.8%^[44]^；另一项随机对照试验发现，延长抗凝预防时间，可使VTE发生率降低约55%^[45]^。Hachey等^[46]^的研究发现，经改良的Caprini风险评估模型，有助于筛选出在延长抗凝治疗中获益的肺癌手术患者。Sterbling等^[47]^则在近期发现，利用指南推荐的风险评估模型，可在不增加出血风险的同时，降低胸部肿瘤手术患者症状性VTE的发生率。

基于以上事实，美国肿瘤学会（American Society of Clinical Oncology, ASCO）2019版《肺癌患者静脉血栓栓塞防治指南》，推荐抗凝从术前开始，术后维持最少7 d-10 d^[48]^；美国胸科医师协会（American College of Chest Physicians, ACCP）第9版《静脉血栓栓塞防治指南》，虽未对抗凝起始时间做准确说明，但对VTE高危肿瘤患者，推荐抗凝预防时间应延长至术后4周^[49]^；美国国家综合癌症网络（National Comprehensive Cancer Network, NCCN）2017版《癌症相关静脉血栓栓塞指南》则建议依据患者VTE风险评分高低与出血风险等级不同，选择使用的药物种类，在术后12 h-72 h内，给予药物抗凝预防，维持7 d-10 d，并在患者出院后继续给予VTE预防治疗，对于极高危患者，预防时间则应相应延长至术后4周^[33]^。综合循证医学证据与指南推荐意见，《共识》建议，使用改良后的Caprini评分量表识别VTE高危的胸部恶性肿瘤患者，抗凝预防措施应在术前12 h开始，术后12 h后继续给予药物抗凝，并持续7 d-10 d；对于极高危患者，抗凝预防应延长至术后30 d，以覆盖围术期VTE发生时间窗。

## 2 国内外VTE预防现状

### 2.1 国外现状

作为接受肿瘤切除手术患者在术后30 d内死亡的最常见与最主要原因，VTE可以通过物理与药物等抗凝措施加以预防^[50]^。然而根据一项国际大型多中心横断面研究^[51]^（ENDORSE研究）的结果，VTE预防情况不容乐观。研究涉及32个国家的68, 183例患者，外科共30, 827例患者及内科共37, 356例患者接受了评估，结果表明，外科患者中，面临VTE发生风险的患者占64.4%，而这其中按照指南推荐进行规范化抗凝预防的患者，仅占58.5%，各国家之间得到规范化抗凝预防的患者比例差值范围为0.2%-92.1%，在研究涉及的亚洲、中东国家，发展中国家及非欧语系国家中，患者得到规范化抗凝预防的比例相对较低。

### 2.2 国内现状

2019年，中国静脉血栓研究组进行了一项大型多中心回顾性研究^[52]^，对2007年-2016年10年范围内国内90家医院的105, 723例住院患者进行统计，发现国内VTE患者住院率从3.2/10万上升至17.5/10万，目前这一数据远远低于西方国家发病率，与韩国、新加坡、中国台湾10年前数据相仿，低于中国香港^[53, 54]^数据；住院VTE患者死亡率从4.7%下降至2.1%，与其他国家相仿。分析认为我国目前VTE发病率远低于真实发病率，这与大量VTE患者未能得到识别有关。随着医疗卫生服务供给的增加、检测设备与技术的进步以及医师对疾病日益加深的认识，我国仍将面临着VTE发生率进一步上升的严峻挑战。

而在2018年国内的另一项多中心横断面研究中，研究者们收集了全国范围内60余家医院13, 609例患者的数据，以ACCP-9指南为标准，发现在其中6, 986例外科手术患者中，有近半数被评价为VTE高危风险，而接受抗凝治疗的外科患者仅占19.0%，按照指南进行规范化抗凝预防的外科患者，更是只占11.8%^[55]^。

《共识》指出，目前我国胸部恶性肿瘤患者接受规范化VTE预防比例较低的主要原因为：医师对VTE认知度和关注度不够；依据以往散发报道，认为VTE发生率较低；担心药物预防后大出血的风险^[1]^。

## 3 小结

胸部恶性肿瘤患者围术期VTE预防是一项需要引起中国胸外科医师重视的问题，此前国际上已有多部针对VTE预防的指南，然而缺乏对胸外科肿瘤患者进行VTE预防的具体化及个体化指导。《共识》的颁布，在提供了一份风险评估、预防、诊断与治疗工具的同时，初步规范了胸部恶性肿瘤围术期VTE的预防。期待以此为契机，今后能够在规范化治疗的基础上出现更多的循证医学证据，为胸外科恶性肿瘤患者带来更加精准化的治疗。
